# 

**Published:** 1910-06

**Authors:** 


					JUNE, 1910.
Vol. XXVIII. No. 108
T THE I LIBBAR\
B RI orF,C?
^ ^ Jul. -i. -?h0
MEDICO-CHIRWRGICAL
PUBLISHED UNDER THE AUSPICES OF
THE BRISTOL MED1CO-CHIRURG1CAL SOCIETT.
Editov: R. SHINGLKTON SMITH, M.D., B.Sc.
WITH WHOM ARE ASSOCIATED
J. MICHELL CLARKE, M.A., M.D.,
J. LACY FIRTH, M.S., M.D., JAMES SWAIN, M.S., M.D.
P. WATSON WILLIAMS, M.D., Assistant Editor.
Editorial Secretary: J. A. NIXON, B.A., M.B.
BRISTOL: J. W. ARROWSMITH.
London : J. & A. Churchill, 7 Great Marlborough Street.

				

